# Supplementary material for: Antibiotic Resistance and Genetic Determinants of Helicobacter pylori in Oman: Insights from Phenotypic and Whole-Genome Analysis
Source: Int J Mol Sci. 2025 Jun 12;26(12):5628. doi: 10.3390/ijms26125628 (PMC12193486; doi:10.3390/ijms26125628)
Supplement: Supplementary file 1 [file ijms-26-05628-s001.zip › ijms-3619707-supplementary.pdf]

Table S1 General features for *H. pylori* genome sequences of local 20 isolates.

Sample Id	#contigs (>=0 bp)	#contigs (>=1000bp)	Total length (>=0bp)	Total length (>=1000bp)	#contigs	Largest contig	Total length	GC (%)	N50	N75	L50	L75	#N's per 100kbp
HP1	40	25	1639646	1634586	28	391172	1636740	38.86	139298	52965	4	8	0
HP 2	38	23	1674836	1669133	26	301382	1671768	38.74	133768	62479	4	9	0
HP 3	30	20	1673696	1670826	21	554354	1671824	38.73	133760	69130	3	7	0
HP 4	310	111	1674899	1610323	139	98981	1629764	38.86	25889	14506	19	41	0
HP 5	35	20	1589006	1585110	21	289144	1586010	38.97	166822	88184	4	7	0
HP 6	99	46	1669297	1653665	53	255035	1658972	38.74	62078	33252	8	18	0
HP 7	63	42	1655431	1648226	46	300140	1651632	38.74	81542	42882	6	13	0
HP 8	164	90	1679401	1650559	109	138942	1663902	38.92	34646	22977	15	30	0
HP 9	70	49	1635665	1628931	53	228667	1631970	38.87	99427	41534	6	13	0
HP 10	41	23	1616499	1611183	25	344735	1613034	39.04	229000	74945	3	6	0
HP11	42	24	1648687	1641586	28	514317	1645184	38.86	138883	60575	4	7	0
HP12	37	29	1646058	1643365	31	229137	1644859	38.8	121746	62947	5	10	0
HP13	28	15	1602598	1597835	18	414400	1600443	38.96	229058	158904	3	5	0
HP14	45	26	1657395	1650401	31	221466	1654176	38.79	136695	72398	5	9	0
HP15	57	32	1641279	1632047	36	417775	1635631	38.97	110410	49134	4	10	0
HP16	162	135	1582971	1567585	153	74869	1579569	38.97	18311	10845	29	57	0
HP17	30	20	1671433	1666827	23	423102	1669629	38.71	199192	61988	3	7	0
HP18	33	23	1653289	1650048	25	414007	1651383	38.81	218001	103637	3	6	0
HP19	32	17	1591057	1586771	17	314848	1586771	39.10	172939	172939	4	6	0
HP20	90	40	1626568	1612635	42	162414	1614254	38.87	74903	53793	8	14	0

Table S2 Sequence types of *H. pylori* isolates.

Isolate	Sequence type (MLST) CGE	HpTT
Oman		
OmanHP1	Novel type	3120
OmanHP 2	Novel type	3120
OmanHP 3	Novel type	3120
OmanHP 4	Novel type	Novel type
OmanHP5	Novel type	3120
OmanHP 6	Novel type	Novel type
OmanHP 7	Novel type	Novel type
OmanHP 8	Novel type	3120
OmanHP 9	Novel type	3120
OmanHP 10	Novel type	Novel type
OmanHP 11	Novel type	Novel type
OmanHP 12	Novel type	3104
OmanHP 13	Novel type	3120
OmanHP 14	Novel type	3120
OmanHP 15	Novel type	3120
OmanHP 16	Novel type	3120
OmanHP 17	Novel type	3120
OmanHP 18	Novel type	3120
OmanHP 19	Novel type	Novel type
OmanHP 20	Novel type	Novel type
Iran		
Iran1	Novel type	3120
Iran2	Novel type	3096
Iran3	Novel type	3120
Iran4	Novel type	Novel type
Iran5	Novel type	3120
Iran6	Novel type	3084
Iran7	Novel type	Novel type

Iran8	Novel type	3120
Iran9	Novel type	3084
Iran10	Novel type	3120
Iran11	Novel type	3084
Iran12	Novel type	3084
Iran13	Novel type	3066
Iran14	Novel type	3100
Iran15	Novel type	3120
Iran16	Novel type	3120
Iran17	Novel type	3120
Iran18	Novel type	3120
Iran19	Novel type	3120
Iran20	Novel type	3120
India		
India1	Novel type	Novel type
India2	Novel type	Novel type
India3	Novel type	Novel type
India4	Novel type	Novel type
India5	Novel type	Novel type
India6	Novel type	Novel type
India7	Novel type	Novel type
India8	Novel type	Novel type
India9	Novel type	Novel type
India10	Novel type	Novel type
India11	Novel type	Novel type
India12	Novel type	Novel type
India13	Novel type	Novel type
India14	Novel type	Novel type
India15	Novel type	Novel type
India16	Novel type	Novel type
India17	Novel type	Novel type
India18	Novel type	Novel type
India19	Novel type	Novel type
India20	Novel type	Novel type

Russia		
Russia1	Novel type	3120
Russia2	Novel type	3120
Russia3	Novel type	Novel type
Russia4	Novel type	Novel type
Russia5	Novel type	Novel type
Russia6	Novel type	3120
Russia7	Novel type	Novel type
Russia8	Novel type	Novel type
Russia9	Novel type	Novel type
Russia10	Novel type	3120
Russia11	Novel type	3120
Russia12	Novel type	Novel type
Russia13	Novel type	3120
Russia14	Novel type	3096
Russia15	Novel type	Novel type
Russia16	Novel type	3120
Russia17	Novel type	3120
Russia18	Novel type	3120
Russia19	Novel type	3100
Russia20	Novel type	Novel type
Germany		
Germany1	Novel type	3066
Germany2	Novel type	Novel type
Germany3	Novel type	Novel type
Germany4	Novel type	3120
Germany5	Novel type	Novel type
Germany6	Novel type	3120
Germany7	Novel type	3120
Germany8	Novel type	Novel type
Germany9	Novel type	Novel type
Germany10	Novel type	Novel type
Germany11	Novel type	3096

Germany12	Novel type	3120
Germany13	Novel type	Novel type
Germany14	Novel type	1233
Germany15	Novel type	3120
Germany16	Novel type	Novel type
Germany17	Novel type	3120
Germany18	Novel type	Novel type
Germany19	Novel type	3120
Germany20	Novel type	3120
